# Integrated Transcriptomic and Metabolomic Analysis of the Mechanism of Intramuscular Fat Differences in Wandong Cattle

**DOI:** 10.3390/ijms262311557

**Published:** 2025-11-28

**Authors:** Fenglou He, Han Liu, Yakun Yao, Zhanhong Qiao, Xinye Li, Chao Chen, Xiaokang Lv, Ke Ji, Jinling Hua

**Affiliations:** College of Animal Science, Anhui Science and Technology University, Fengyang 233100, China; 18889340981@163.com (F.H.); lh1525672@163.com (H.L.); 16652015227@163.com (Y.Y.); 18731310109@163.com (Z.Q.); 14792810387@163.com (X.L.); chenchao@ahstu.edu.cn (C.C.); lvxk@ahstu.edu.cn (X.L.)

**Keywords:** wandong cattle, intramuscular fat content, transcriptomics, metabolomics

## Abstract

This study aimed to collaboratively investigate the mechanism of variations in intramuscular fat (IMF) content in Wandong cattle using transcriptomics and metabolomics techniques. Longissimus dorsi (LD) muscle samples were collected from thirteen free-range Wandong cattle in Fengyang County, Anhui Province, China. From this initial cohort, eight animals closely matched in age and body weight were selected. Based on IMF content measured by Soxhlet extraction, these eight cattle were divided into two groups: the high-IMF (HF, n = 4) and low-IMF (LF, n = 4) groups. Subsequent analyses were performed on integrated datasets comprising the transcriptome, metabolome, and fatty acid profile. The results revealed a significant increase in IMF in the HF group compared to the LF group (*p* < 0.05). Specifically, α-linolenic acid (C18:3n3) and γ-linolenic acid (C18:3n6) were significantly more abundant in the LF group compared to the HF group (*p* < 0.05), whereas oleic acid (C18:1n9c) and cis-9-palmitoleic acid (C16:1) predominated in the HF group. However, saturated fatty acids (SFAs), such as myristic acid (C14:0), palmitic acid (C16:0), stearic acid (C18:0), and Margaric acid (C17:0), did not show significant differences (*p* > 0.05). A total of 9164 differentially expressed genes (DEGs) were identified via transcriptome analysis, with 2202 genes upregulated and 6962 genes downregulated in the HF group compared to the LF group. The expression profiles exhibited a distinct pattern, characterized by the upregulation of genes such as FABP1, SREBF1, and LIPE, while genes including SCD, PPARGC1A, and LEP were downregulated. GO enrichment analysis demonstrated that the majority of DEGs were predominantly abundant across 25 distinct functional categories distributed across the three primary ontologies. KEGG pathway analysis further identified 341 significantly enriched signaling pathways in the HF group (*p* < 0.05), predominantly involving metabolic pathways, FoxO, AMPK, and PPAR signaling pathways. Untargeted liquid chromatography-tandem mass spectrometry (LC-MS/MS) metabolomics analysis revealed 404 differential accumulated metabolites (DAMs), with 187 in positive ion mode and 217 in negative ion mode (*p* < 0.05). These DAMs were notably enriched in pathways such as glycerophospholipid metabolism, terpene and steroid biosynthesis, fatty acid degradation, and fatty acid metabolism. Notably, C16:1, C18:1n9c, arachidonic acid (peroxide free) (C20:4n6), oleoyl-L-carnitine, and linoleoyl-carnitine were identified as key players in lipid metabolism. Integrating transcriptomics with metabolomics data unveiled significant associations between DAMs linked to lipid metabolism and DEGs. Specifically, C18:1n9c exhibited a positive correlation with LPIN3, while C16:1 showed negative associations with PPAP2B, PPAP2A, CDS2, HADHA, LPL, HSD17B12, ELOVL5, ACSL1, and ACOX1, and positive correlations with PLA2G15, CDIPT, AGPSBG1, and GPD1. In summary, the variation in IMF content in Wandong cattle is co-regulated by key genes (SREBF1, ACSL1, SCD) via the AMPK, PPAR, and FoxO signaling pathways, coupled with alterations in specific fatty acid metabolites such as C18:1n9c, C16:1, and C20:4n6. These findings provide critical molecular insights for the genetic selection and breeding of Wandong cattle, which are renowned for their superior meat quality.

## 1. Introduction

With the improvement of living standards, the demand for high-quality beef is increasing [1]. Intramuscular fat (IMF) content is a crucial indicator of meat quality, and plays a significant role in determining the sensory characteristics of meat, such as flavor, tenderness, and juiciness [2,3]. Studies have shown that IMF content is influenced by a complex interplay of cellular and metabolic factors, such as the proliferation and differentiation of adipose precursor cells and the regulation of lipid metabolic homeostasis [4,5]. Schettini et al. [6] identified that genes like ECHS1 and NFIA affect IMF content in Nellore cattle by regulating linoleic acid and α-linolenic acid metabolic pathways. Frezarim et al. [7] utilized multi-omics integration to demonstrate that CAND1, ACTN4, FGFR2, and NCOR2 function as a regulatory hub that couples cytoskeletal remodeling with lipid-synthetic pathways to orchestrate IMF in Nellore cattle. These findings underline the crucial impact of gene regulation on IMF content, thus providing a valuable reference point for breeding high-quality beef. The significant demand for high-quality beef in China has led to a rapid transition of indigenous cattle breeds, such as Qinchuan [8], Jinnan [9], Nanyang [10] and Luxi [11], from service beef cattle utilization to specialized beef production. It is imperative to comprehend the molecular mechanisms that govern the process of IMF deposition in order to enhance the quality of beef. Some studies have shown that the IncFABP4 factor promotes adipocyte differentiation in water buffalo muscle tissue by upregulating regulators such as PPARD and C/EBPα, thereby increasing IMF accumulation [12]. Ma et al. [13] demonstrated that ALDOC activates the AKT-mTORC1 pathway to stimulate IMF deposition in pigs, suggesting its potential as a target for meat quality improvement. Notably, Fu et al. [14] found that dietary addition of betaine up-regulated lipogenesis and down-regulated abundance of genes associated with lipolysis, improving meat quality and IMF content in pigs. Arginine has been shown to regulate lipid metabolism and meat quality in lambs through the gut microbiota-mediated SCFAs/GLP-1/GLP-1R/AMPK signaling pathway [15]. These studies have demonstrated the ability to enhance meat quality by modulating key lipogenic and metabolic pathways. Wandong cattle, a distinguished local yellow cattle breed in Anhui Province, recognized as a superior local livestock resource by the China Poultry Genetic Resource Committee, holds a ‘3A+’ grade [16,17]. While historically utilized as a dual-purpose breed for draft service and meat production, Wandong cattle are now being developed as a specialized beef breed [18]. These animals are characterized by their robust constitution, adaptability, and efficiency of digestion of roughage. The shearing force of Wandong cattle is well-recognized, with a shear force value (30.87 ± 5.06 N) rivaling that of Angus cattle (40.09 ± 6.67 N) [18,19]. Despite the well-documented tenderness of their meat and favorable profile of unsaturated fatty acids, there remains a discrepancy between the meat’s quality and the increasing consumer demand for consistently high-quality beef. Consequently, further genetic enhancement and targeted breeding strategies are imperative to optimize its meat quality traits. Research on the quality traits of Wandong cattle meat is currently limited, with only a few studies investigating the association between polymorphisms in the CAPN1 and CAST genes and meat quality attributes [20]. It is imperative that more profound and exhaustive studies are conducted to elucidate the underlying genetic mechanisms and identify additional pivotal genes that influence IMF deposition. In considering the intricate nature of IMF deposition, integrated transcriptomic and metabolomic approaches offer distinct advantages for elucidating its molecular mechanisms. Transcriptomics facilitates the systematic identification of key regulatory genes and signaling pathways involved in adipogenesis and lipid metabolism within muscle tissue, while metabolomics captures the dynamic end products of metabolic flux. For instance, Zhang et al. [21] employed integrated transcriptomic and metabolomic analyses to identify FADS2, ACOT7, and ACOT2 as key regulators of fatty acid metabolism in cattle, and demonstrated that a 46-amino acid deletion in the transmembrane domain of FADS2 alters lipid metabolism, thereby promoting IMF deposition. These findings indicate that integrating these two omics layers is imperative for constructing a comprehensive regulatory network from gene expression to metabolic function, thereby providing a holistic understanding of IMF deposition.

Consequently, this study aims to comprehensively analyze the molecular mechanism of IMF deposition in Wandong cattle by utilizing transcriptomic and metabolomic technologies. The research will identify key regulatory genes and metabolic pathways to establish a theoretical foundation and practical targets for molecular breeding and the selection of high-quality beef cattle.

## 2. Results

### 2.1. The Contents of Lipid and Fatty Acid in LD of Wandong Cattle

The HF group displayed significantly higher IMF content (17.42) compared to the LF group (10.73; *p* = 0.031), accompanied by distinct fatty acid profiles. There were notable differences in the profile of fatty acids between the two groups. Importantly, in the HF group, the content of polyunsaturated fatty acids (PUFAs) was lower than that in the LF group. Specifically, α-linolenic acid (C18:3n3) levels were significantly reduced in HF cattle (0.53 vs. 1.27; *p* = 0.015), while γ-linolenic acid (C18:3n6) displayed a marked decreasing trend (0.20 vs. 0.39; *p* = 0.041). In contrast, monounsaturated fatty acids (MUFAs) were significantly higher in the HF group. The analysis indicated a statistically significant difference in total MUFAs levels between the two groups (*p* =0.038), with the HF group averaging 40.93 and the LF group averaging 30.19. Specifically, the HF group showed significantly higher levels of cis-9-palmitoleic acid (C16:1) at 3.90 compared to the LF group’s 2.37 (*p* = 0.049) and oleic acid (C18:1n9c) at 37.03 compared to 27.83 (*p* = 0.049). Significantly, while MUFAs and partial PUFAs differed, the total saturated fatty acids (SFAs) and total PUFAs did not exhibit statistically significant differences between the two groups (*p* > 0.05). Specifically, various individual fatty acids demonstrated similar levels across the groups, including saturated fatty acids such as myristic acid (C14:0), palmitic acid (C16:0), margaric acid (C17:0), and stearic acid (C18:0); as well as unsaturated species such as linoleic acid (C18:2n6c), dihomo-γ-linolenic acid (C20:3n6), arachidonic acid (peroxide free) (C20:4n6), and eicosapentaenoic acid (C20:5n3) (Table 1).

### 2.2. RNA Sequencing and Identification of DEGs in LD of Wandong Cattle

In this study, RNA sequencing technology was used on the gene expression disparities in the longissimus dorsi (LD) muscle tissues of the HF (H1, H2, H3) and LF (L1, L2, L3) groups of Wandong cattle. The findings demonstrated that the LF and HF groups possessed sequencing data ranging from 45, 722, 634 to 48, 466, 588 and 42, 850, 892 to 57, 277, 954, respectively. The GC content of the data was predominantly around 50%, and the Q30 values were all above 93.02% (Table 2). Principal component analysis (PCA) was employed to reveal that the first principal component (PC1) and the second principal component (PC2) accounted for 90.9% and 9% of the total variance, respectively.

**Figure 1:**
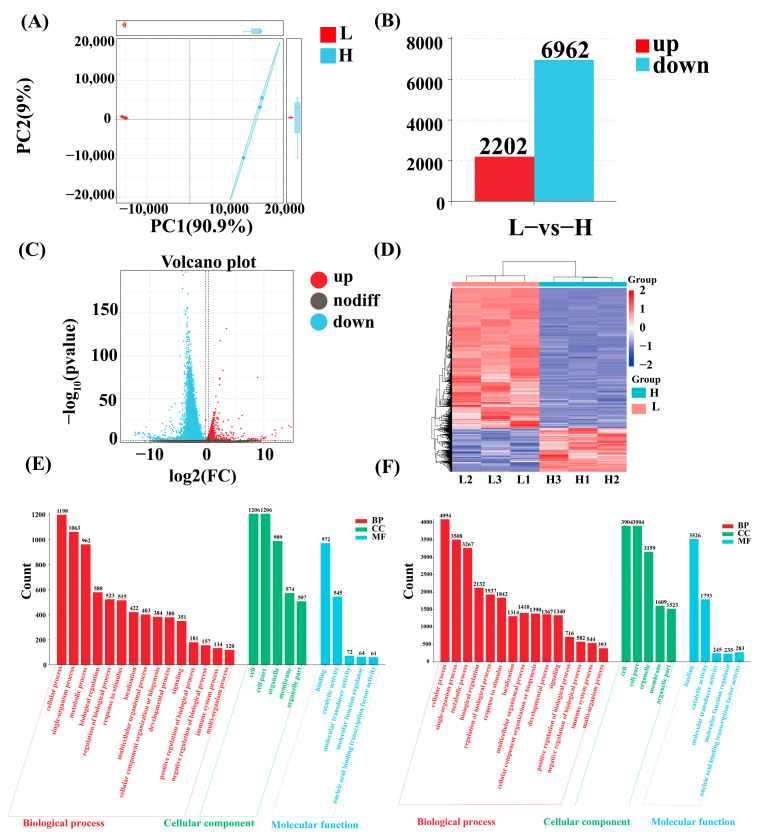
Differentially expressed genes in the HF group and LF group and GO enrichment analysis of differentially expressed genes. (**A**) PCA score plot of transcriptomes. (**B**) Histogram of differential gene expression statistics. (**C**) Volcano plot of differentially expressed genes. (**D**) Subgroup clustering analysis; red in the figure represents highly expressed genes, and blue represents lowly expressed genes. (**E**) GO enrichment of upregulated genes: biological processes (red, top 15), cellular components (green, top 5), and molecular functions (blue, top 5). (**F**) GO enrichment of downregulated genes: biological processes (red, top 15), cellular components (green, top 5), and molecular functions (blue, top 5).

The HF and LF groups exhibited distinct clusters (Figure 1A). A total of 9164 differentially expressed genes (DEGs) (FC ≥1.2, *p* < 0.05) were detected, with 2202 genes up-regulated and 6962 genes down-regulated in the HF group compared to the LF group (Figure 1B,C). Hierarchical clustering results indicated significant differences in the expression profiles of genes between the two groups (Figure 1D). GO enrichment analysis was performed to identify the DEGs that were significantly enriched in 25 items (*p* < 0.05). The significantly enriched biological processes were mainly related to biological regulation and the regulation of biological process, etc.; the significantly enriched molecular functions were mainly related to binding and catalytic activity, etc.; and the significantly enriched cellular components were mainly related to cell, cell parts, etc. (Figure 1E,F).

**Figure 2:**
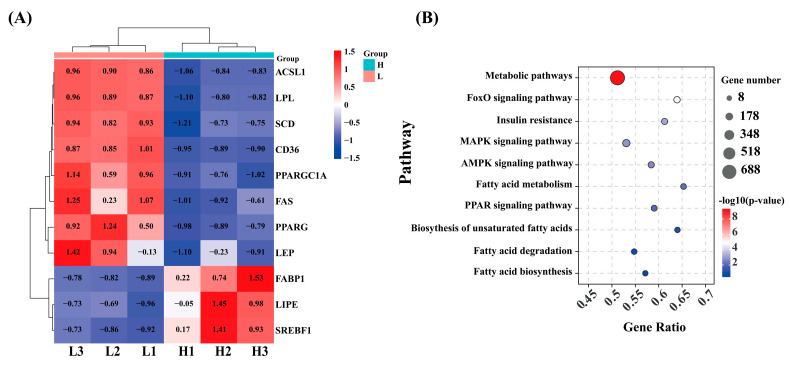
Analysis of differential genes associated with lipid metabolism. (**A**) Cluster analysis heatmap. Red in the figure represents highly expressed genes, and blue represents lowly expression genes. (**B**) KEGG pathway enrichment analysis. The size of the circle corresponds to the number of genes enriched in the pathway, and the color intensity represents the -log_10_ (*p*-value), with red indicating higher statistical significance.

KEGG pathway analysis revealed that the DEG were enriched in 341 pathways. During these DEGs, some genes related to lipid metabolism were quantified, such as PPARGC1A, PPARG, SCD, SREBF1, CD36, LIPE, LEP, FAS, LPL, ACSL1, and FABP1 (Figure 2A). These genes primarily participated in signaling pathways, including the AMPK signaling pathway (ko04152), MAPK signaling pathway (ko04010), PPAR signaling pathway (ko03320), cholesterol metabolism (ko04979), and other metabolic pathways (Figure 2B).

### 2.3. Metabolite Data Analysis in IMF of Wandong Cattle

**Figure 3:**
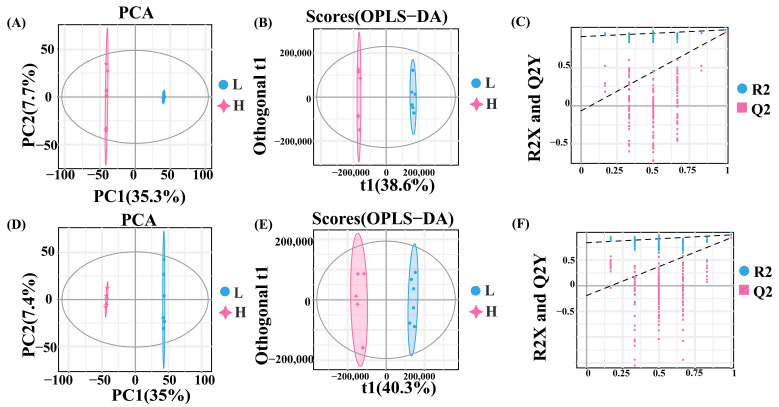
PCA and OPLS-DA of metabolites. (**A**) PCA score plot of metabolites in positive ion mode. (**B**) OPLS-DA used for multivariate statistical analysis in positive ion mode. (**C**) Permutation plot, in positive ion mode. The load range is −1 to 1. A load close to −1 or 1 indicates a strong influence of the variable on the component, while a load close to 0 indicates a weak influence. (**D**) PCA score plot of metabolites in negative ion mode. (**E**) OPLS-DA used for multivariate statistical analysis in negative ion mode. (**F**) Permutation plot in negative ion mode. The load range is −1 to 1. A load close to −1 or 1 indicates a strong influence of the variable on the component, while a load close to 0 indicates a weak influence.

Analysis of the metabolomic data demonstrated that PCA revealed distinct intra-group clustering and inter-group separation: HF and LF samples were segregated in both positive ion (PC1: 35.3%, PC2: 7.7%; Figure 3A) and negative ion modes (PC1: 35.0%, PC2: 7.4%; Figure 3D). Orthogonal partial least squares-discriminant analysis (OPLS-DA) modeling was employed to identify differential accumulated metabolites (DAMs), with permutation tests confirming significant group discrimination (*p* < 0.05). The positive-ion model yielded R^2^X = 0.514 and R^2^Y = 0.99 (Figure 3B,C), while the negative-ion model produced R^2^X = 0.566 and R^2^Y = 0.991 (Figure 3E,F).

**Figure 4:**
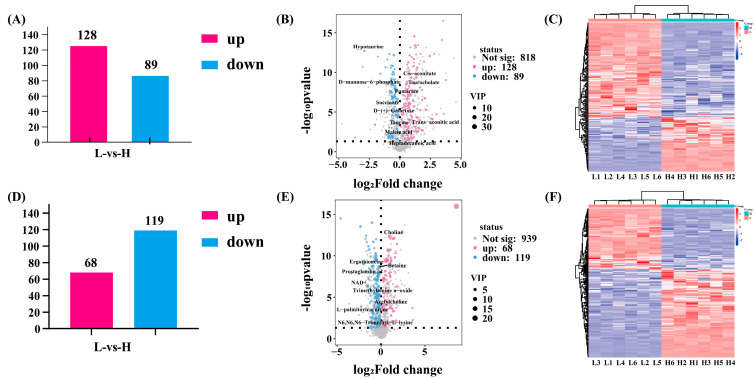
Comparative analysis of metabolite alterations in positive and negative ion modes. Panels (**A**–**C**) and (**D**–**F**) display the results from the negative and positive ion modes, respectively. (**A**,**D**) Histogram of significantly altered metabolites showing upregulated (red) and downregulated (blue) counts (threshold: |log_2_FC|> 1, *p* < 0.05). (**B**,**E**) Volcano plots of metabolic fold-change (log_2_FC) against statistical significance (-log_10_(*p*-value)). Metabolites were considered significantly altered if they exceeded the thresholds of |log_2_FC| > 1, *p* < 0.05, and a VIP score > 0.8. (**C**,**F**) Hierarchical clustering heatmaps display the relative abundance patterns of the significantly differential metabolites across samples.

Distinct metabolic profiles between HF and LF groups were thereby established. In the negative ion mode, 1035 metabolites were identified, with 217 exhibiting significant abundance discrepancies between high and low frequency samples; among these, 128 were up-regulated and 89 were down-regulated (Figure 4A,B). The permutation test results in negative ion mode demonstrate that the original model comparing the high and low groups is robust and exhibits no signs of overfitting (Figure 4C). In the positive ion mode, 1126 metabolites were detected, with 187 showing significant differences in abundance between high and low frequency samples. Specifically, 119 were up-regulated and 68 were down-regulated (Figure 4D,E). Similarly, the permutation test conducted in the positive ion mode corroborated the findings of the original model, demonstrating that it exhibited robust separation between the high and low groups, with no indication of overfitting (Figure 4F).

**Figure 5:**
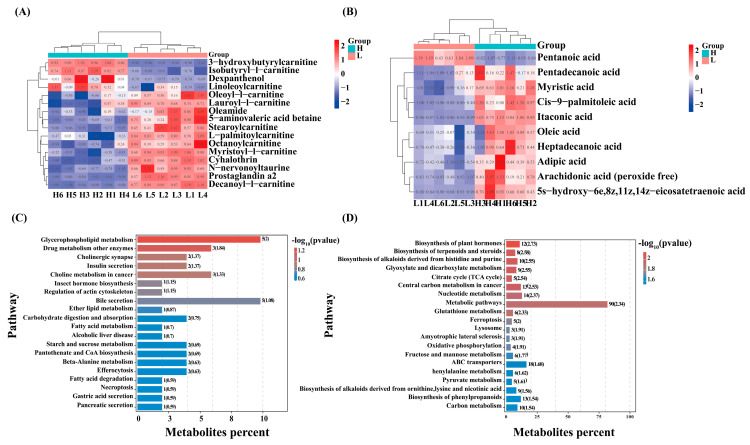
KEGG enrichment analysis of differentially expressed metabolites with lipid metabolism. (**A**) Differential metabolites related to lipid metabolism in positive ion mode. (**B**) Differential metabolites related to lipid metabolism in negative ion mode. (**C**) KEGG pathway enrichment analysis of differentially abundant metabolites identified in positive ion mode. (**D**) KEGG pathway enrichment analysis of differentially abundant metabolites identified in negative ion mode.

Among all the detected metabolites, the results showed that there were 26 DAMs associated with lipid metabolism, of which 16 were found in the positive ionic mode, mainly including L-palmitoylcarnitine and oleoyl-L-carnitine, etc. (Figure 5A). 10 were found in the negative ionic mode, mainly including C20:4n6, C16:1, and Oleic acid, etc. (Figure 5B). Enrichment by KEGG showed that DAMs in the positive mode pattern were significantly enriched in glycerophospholipid metabolism, ether lipid metabolism, carbohydrate digestion and absorption, fatty acid metabolism, and fatty acid degradation (Figure 5C). In the negative ion mode (Figure 5D), the DAMs enriched pathways including the biosynthesis of terpenoids and steroids, the citrate cycle (TCA cycle), fructose and mannose metabolism, ABC transporters, and carbon metabolism.

### 2.4. Integrated Analysis of DEGs and DAMs in IMF of Wandong Cattle

**Figure 6:**
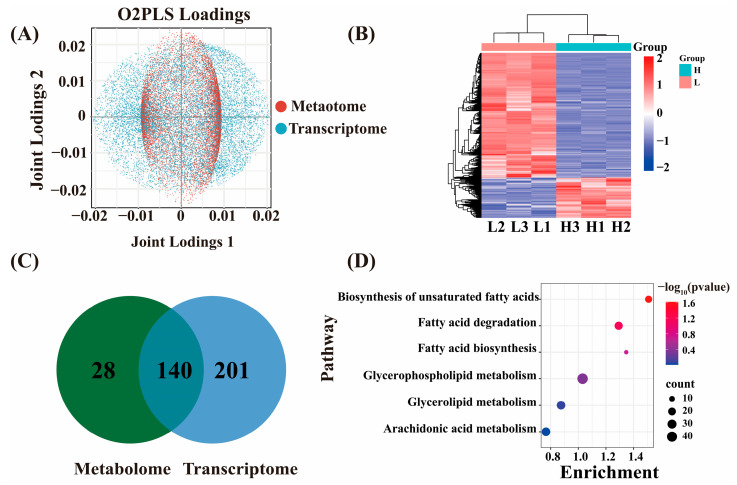
Joint metabolomic and transcriptomic correlation analysis. (**A**) O2PLS analysis. (**B**) Correlation heat map of transcripts and metabolites. (**C**) Venn diagram of shared KEGG terms between transcriptome and metabolome. (**D**) Shared KEGG terms among transcriptome and metabolome.

**Figure 7:**
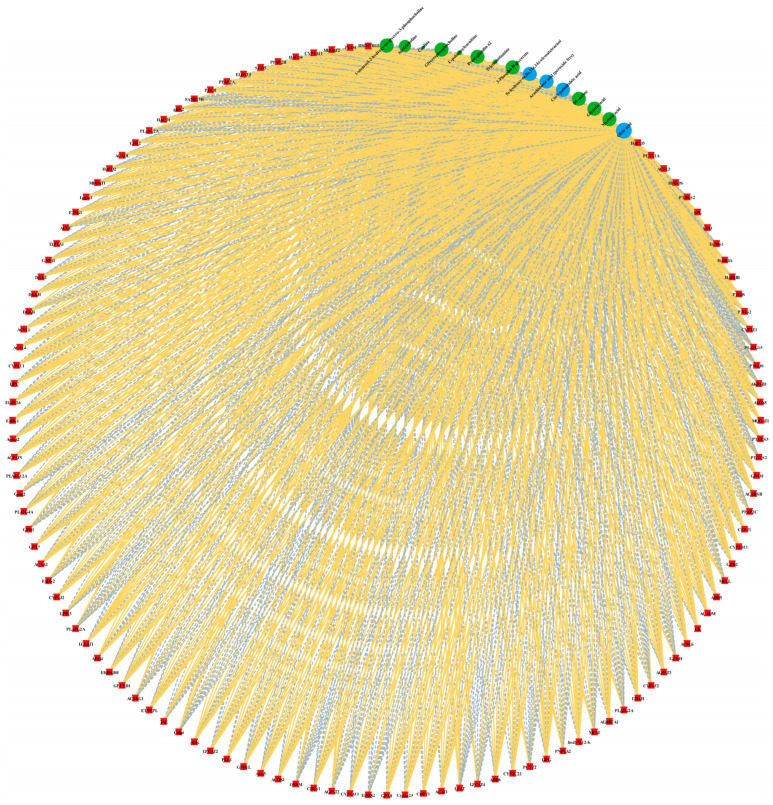
Correlation network of differentially expressed genes and differential metabolites in the integrated pathway with red nodes representing genes, green and blue nodes indicating metabolites, yellow lines showing significant positive correlations, and gray lines denoting significant negative correlations.

In order to resolve the coupling between the transcriptome and metabolome, a bidirectional O2PLS model was constructed. The O2PLS joint loading plot revealed a synergistic distribution of transcriptome and metabolome variables on the shared components, and the clustering of the HF and LF group samples was clearly distinguished, which suggests that the two histologies are tightly related (Figure 6A). Correlation analysis and clustering of genes and metabolites from different samples revealed a significant clustering trend between the HF and LF group samples (Figure 6B). Venn diagram analysis showed 140 KEGG pathways to be jointly enriched by the transcriptomic and metabolomic datasets, whereas 201 and 28 pathways were uniquely captured by the transcriptome and metabolome, respectively (Figure 6C). Among the shared pathways, six displayed significant enrichment (*p* < 0.05) and were directly related to lipid metabolism: fatty acid biosynthesis, fatty acid degradation, biosynthesis of unsaturated fatty acids, glycerophospholipid metabolism, glycerolipid metabolism, and arachidonic acid metabolism (Figure 6D). The DEGs and DAMs involved in these pathways were subsequently overlaid on the corresponding KEGG network diagrams (Figure 7). In the unsaturated fatty acid biosynthesis pathway, SCD, SCD5, ACOX1, ELOVL5, HSD17B12, and others were identified as DEGs, whereas C18:1n9c and C20:4n6 were detected as the principal DAMs. For fatty acid biosynthesis, ACSL4, ACSL6, and ACSL1 were found to be differentially expressed, with C16:1, C14:0, and C18:1n9c serving as the key discriminant metabolites. Within fatty acid degradation, 23 genes—including ACSL3, HADHB, ACSL6, ACOX1, ACSL5, ACAA2, ACSL4, ACSL1, and others—were differentially expressed, with l-palmitoylcarnitine identified as the primary metabolite marker. In the arachidonic acid metabolism signaling pathway, 27 genes—among them HPGDS, PLA2G2A, FAM213B, and others—were found to be differentially expressed, alongside Prostaglandin a2, 5s-hydroxy-6e,8z, 11z,14z-eicosatetraenoic acid, and C20:4n6 as the principal metabolites. Within glycerolipid metabolism, 26 genes—including PPAP2C, LIPG, LPIN3, MBOAT1, PPAP2A, PPAP2B, and others—were differentially expressed, with 3-phospho-D-glycerate and glyceric acid representing the key metabolites. Finally, in glycerophospholipid metabolism, 45 genes—among them, PPAP2C, GPD2, LPIN3, GPD1, MBOAT1, CDS2, PPAP2A, PPAP2B, MBOAT2, and more—were differentially expressed, together with 1-stearoyl-2-hydroxy-sn-glycero-3-phosphocholine, acetylcholine, choline, glycerophosphocholine, triethanolamine, and dl-serine as the primary discriminant metabolites (Table 3).

**Figure 8:**
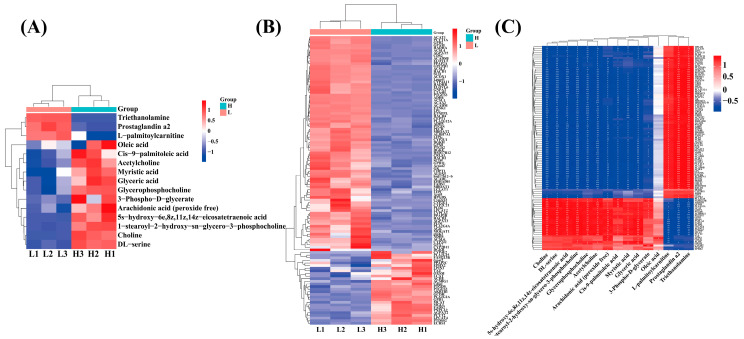
Correlation analysis of lipid metabolism-related genes and metabolites and their expression. (**A**) Heat map of lipid metabolism-related metabolites. (**B**) Heat map of lipid metabolism-related genes. (**C**) Correlation analysis of fatty acid-related genes and metabolites.* *p* < 0.05, ** *p* < 0.01 and *** *p* < 0.001.

Analysis of the genes and metabolites involved in the six significantly different KEGG signaling pathways jointly enriched by the transcriptomic and metabolomic datasets revealed pronounced, directional modulation of key metabolites within the LD of Wandong cattle. Relative to the LF group, triethanolamine, prostaglandin a2 and l-palmitoylcarnitine were significantly downregulated (Figure 8A). Conversely, oleic acid, cis-9-palmitoleic acid, acetylcholine, c14:0, glyceric acid, glycerophosphocholine, 3-phospho-d-glycerate, arachidonic acid (peroxide free), 5s-hydroxy-6e,8z,11z,14z-eicosatetraenoic acid, 1-stearoyl-2-hydroxy-sn-glycero-3-phosphocholine, choline, and dl-serine were significantly upregulated (Figure 8A). In the HF of Wandong cattle, marked transcriptional reprogramming was observed; ACSL1, ACSL3, ACSL4, ACSL6, SCD, SCD5, FADS1, FADS2, ELOVL5, ELOVL6, HADHA, ACOX1, LPL, MBOAT1, and additional loci were significantly down-regulated, whereas CYP4F2, PLA2G15, GPAT2, PPAP2C, EPHX2, LPIN3, TAZ, LCAT, ACHE, ACSBG1, PTGDS, PTGES2, GCDH, PLA2G2A, GPD1, MGLL, FADS2, PNPLA2, AGPAT2, PCYT2, LPCAT4, PTDSS2, ECHS1, and further genes were significantly up-regulated (Figure 8B). Pearson correlation analyses (|r| > 1, *p* < 0.05) were performed between metabolites and genes mapped to fatty acid biosynthesis and the biosynthesis of unsaturated fatty acid. C18:1n9c was significantly positively correlated with LPIN3; metabolites spanning five additional KEGG pathways—namely choline, dl-serine, 5s-hydroxy-6e,8z,11z,14z-eicosatetraenoic acid, 1-stearoyl-2-hydroxy-sn-glycero-3-phosphocholine, glycerophosphocholine, acetylcholine, arachidonic acid (peroxide free), cis-9-palmitoleic acid, glyceric acid, and 3-phospho-d-glycerate—were significantly negatively correlated with PPAP2B, PPAP2A, CDS2, HADHA, LPL, HSD17B12, ELOVLS, ACOX1, etc. Meanwhile, positive correlations were observed with PLA2G15, CDIPT, ACBSBG1, GPD1, etc.; l-palmitiylcarnitine, prostaglandin a2, and triethanolamine were significantly negatively correlated with AKR1B1, GCDH, PLA2G2A, LCAT, CDIPT, PTGDS, ACBSBG1, PTGES2, GDI1, MGLL, PNPLA2, FADS2, ECHS1, LPCAT4, AGPAT2, PDSS2, MCAT, and PCYT2, yet were significantly positively correlated with ELOVLS, PPAP2B, HADHA, CDS2, LIPG, MBOAT2, PPAP2A, ALOX5, ELOVL6, SCD5, SCD, ACAA2, ACOX1, LPL, etc. (Figure 8C).

## 3. Discussion

IMF content is a critical determinant of meat quality [22]. This association between IMF content and the fatty acid profile has been observed across species such as cattle [23] and sheep [24], suggesting the existence of a conserved biological mechanism. Our systematic examination of the HF and LF groups revealed a significant increase in MUFAs and a relative decrease in polyunsaturated fatty acids (PUFAs) in the HF group. This trend aligns with the MUFA enrichment documented in high IMF by Jeong et al. [25]. Additionally, Dávila-Ramírez et al. [26] noted that a reduction in lamb IMF was associated with lower MUFA levels, further supporting our findings. Specifically, we observed a significant enrichment of C18:1n9c and C16:1, accompanied by a notable reduction in C18:3n3 and C18:3n6 in the HF group. This compositional profile is consistent with studies conducted by Realini et al. [27] and Huang et al. [28]. The effect of IMF content on fatty acid profiles may arise from a more rapid accumulation of MUFAs relative to PUFAs as intramuscular adiposity increases [28], a trend that aligns with our findings. Although factors such as age, sex, and diet may affect IMF deposition, all experimental animals in this study were raised under uniform grazing conditions and shared similar biological backgrounds. Consequently, the observed inter-group differences are likely due to intrinsic molecular regulatory mechanisms rather than external environmental influences. This perspective is supported by prior research. For example, a study demonstrated that supplementing pigs’ diets with isoleucine could suppress AMPK phosphorylation and enhance the expression of lipid-related genes such as SCD, FAS, and FABP4, leading to elevated levels of both MUFAs and IMF [29]. This underscores the crucial function of genes like SCD1, which facilitate the conversion of SFAs into MUFAs. A deeper understanding of these molecular mechanisms could ultimately enhance the meat quality of Wandong cattle through targeted exploration of IMF regulation.

In addition, given intricate regulation of IMF deposition by a network of genes and signaling pathways [30,31], the present study conducted a thorough analysis of the transcriptomic profiles of the two groups in question. Transcriptomic analysis identified 9164 DEGs between the HF and LF groups, with 2202 being up-regulated and 6962 down-regulated. Among these DEGs, certain key genes associated with lipid metabolism displayed distinct expression patterns, suggesting the potential direct regulation of IMF deposition and the fatty acid profile. The SREBF1 binds sterol-regulatory elements in target promoters and governs the synthesis of fatty acids, phospholipids, and triglycerides [32,33,34]. FABP1, a liver-specific fatty acid-binding protein, is crucial in regulating lipid storage and distribution [35]. FABP1 primarily facilitates bovine fat accumulation by promoting increased synthesis and release of triglycerides and very-low-density lipoproteins [36]. ACSL1 catalyzes the conversion of long-chain fatty acids to acyl-CoA esters in bovine adipocytes, thereby promoting triglyceride synthesis and adipogenesis [37,38]. LEP-induced activation of AMPK via CAMKK2 results in the suppression of adipogenesis genes and a concomitant reduction in IMF [39]. Deng et al. [40] found that differences in IMF deposition in yaks were attributed to lipid metabolism genes such as SFRP4, FABP4, GADD45A, PDGFRA, RBP4, and DGAT2. Zhao et al. [41] similarly demonstrated that ACSL1 promotes the synthesis of PUFAs and up-regulates the expression of PPARγ, FABP3, ACLY, SCD1, and FASN. This suggests that the elevated levels of PUFAs observed in the LF group compared to the HF group in this study may be attributable to the up-regulation of lipid metabolism genes by ACSL1. Moreover, SCD1 silencing decreases triglycerides, cholesterol, and the desaturation index, and down-regulates SREBF1, FAS, FABP3, and FABP4. These findings suggest that variations in IMF are associated with the genes involved in adipocyte differentiation, lipid storage, and utilization. Cheng et al. [42] and Samovski et al. [38] found that SREBF1, PPARG, and CD36 were co-expressed within the AMPK and PPAR signaling pathways, influencing IMF content by promoting anabolic processes and inhibiting catabolic pathways. Tian et al. [43] also mechanistically confirmed that berberine activates AMPK, suppresses SREBF1 and SCD1, and consequently inhibits cholesterol synthesis and hepatic steatosis in mice. Conversely, elevated ACSL1 expression impairs fatty-acid β-oxidation via PPARγ and enhances triglyceride accumulation [44]. These findings collectively suggested that the deposition of IMF and the variation in the fatty acid profile in Wandong cattle are co-regulated by a network centered on key lipid metabolism genes via AMPK/PPAR signaling pathways.

Metabolomic profiling identified 404 DAMs. Within the 26 lipid-related DAMs identified, crucial UFAs such as MUFAs C16:1 and C18:1n9, as well as the PUFA C20:4n6, were notably increased in the HF group. The elevated levels of circulating MUFAs (C18:1n9c and C16:1) and PUFAs (C20:4n6/C18:3n6) are well-known for their cardioprotective properties in humans [45]. C16:1 is a colorless, water-insoluble lipid factor that effectively modulates systemic lipid metabolism [46]. Feyera et al. [47] demonstrated that increased C16:1 raises plasma triglyceride concentrations in growing piglets, thereby enhancing growth and cold tolerance. C18:1n9c stabilizes lipid droplets; its proportion is significantly higher in the LD muscle of boars than in other adipose depots [48] and it stimulates intramyocardial lipogenesis in cattle [46]. Mechanistically, C18:1n9c activates PPARα, promotes lipolysis and ameliorates hepatic steatosis in Angus bulls [49]. Furthermore, the incorporation of 0.05% C20:4n6 into the parental diet has been shown to optimize the serum lipid profile and liver fatty acid profile of the descendant; this is achieved through the regulation of lipid metabolism-related pathways, significant inhibition of liver fat deposition, and maintenance of lipid homeostasis and metabolic health [50]. The findings of this study indicate that C16:1, C18:1n9c, and C20:4n6, among other metabolites, function as key fatty acids involved in the maintenance of lipid homeostasis through the lipid metabolism pathway in Wandong cattle.

Integrative transcriptomic and metabolomic profiling identified 140 differentially regulated metabolic pathways, among which several key lipid metabolic pathways were prominent. Specifically, the results of the study indicated that C18:1n9c was enriched in the fatty acid biosynthesis and biosynthesis of unsaturated fatty acids pathways. Furthermore, C20:4n6 is predominantly concentrated in the biosynthetic pathway of unsaturated fatty acids. This 20-carbon polyunsaturated fatty acid, characterized by four double bonds, is crucial for maintaining the fluidity and flexibility of biological cell membranes. Notably, C20:4n6 exhibits a strong inverse relationship with the ACSL family, which is primarily involved in fatty acid metabolism, degradation, and biosynthesis pathways. Previous research has shown that polyunsaturated fatty acids, including C20:4n6, serve as substrates for isoforms like ACSL1 and ACSL4, playing a critical role in lipid peroxidation reactions [51]. These findings suggest that the ACSL family may facilitate the catalysis of polyunsaturated fatty acids such as C20:4n6, thereby activating signaling pathways related to fatty acid degradation. Consequently, this process leads to fatty acid oxidation, influencing variations in beef texture and fat content in Wandong cattle. Concurrently, a significant positive correlation was observed between C18:1n9c and LPIN3, a key regulator of lipocalin known for its role in Mg^2+^-dependent phosphatidic acid phosphatase activity in lipid metabolism [52]. LPIN3, functioning as a PPAR transcription factor, has been demonstrated to enhance adipose tissue differentiation and fat deposition in sheep [53]. Our study highlights the predominant association of LPIN3 with glycerophospholipid and glycerolipid metabolic pathways, indicating a potential mechanism by which C18:1n9c may stimulate transcription factors to upregulate LPIN3 expression. Elevated levels of LPIN3, in turn, play a crucial role in modulating C18:1n9c metabolism and promoting lipid synthesis and storage through its pivotal functions in these metabolic pathways. C16:1 exhibited a significant negative correlation with adipose regulators, including LIPC, the ACSL family, SCD1, ELOVL6, FADS1, and ACOX1. This led to the hypothesis in this study that elevated C16:1 levels would decrease the expression of these genes, consequently impacting the accumulation of MUFAs. This observation contrasts with the findings of Zhang et al. [54] in goats, suggesting a potential species-specific regulatory mechanism. Notably, the SCD1 and SCD genes play a role in lipid homeostasis through the AMPK signaling pathway. Acting as an upstream gene of SCD, SREBF1 is also enriched in the AMPK signaling pathway and participates in the regulation of lipid synthesis. It was postulated that SREBF1 would exhibit a negative correlation with C16:1 and regulate lipid synthesis-related genes downstream by inhibiting their expression. De et al. [55] demonstrated that in mice on a high-fat diet, C16:1 enhances glucose uptake and inhibits lipid synthesis by promoting AMPK phosphorylation, increasing glucokinase expression, and reducing SREBF levels. The implication here is that C16:1 potentially modulates lipid metabolism by serving as a mediator in the AMPK signaling pathway to regulate adipokines like SCD, a downstream target of SREBF1. However, additional investigation is necessary to elucidate the precise mechanism in Wandong cattle and to explore the involvement of SREBF1 in the C16:1 mediated lipid metabolism regulation more comprehensively. Essentially, the differences in IMF content in Wandong cattle may be related to specific fatty acids such as C18:1n9c and C16:1. Through integrated analysis, it was found that key signaling pathways such as PPAR and AMPK affected lipid metabolism processes such as fatty acid degradation and glycerophospholipid metabolism by mediating the expression of lipid metabolization-related genes such as SREBF1, SCD, and ACSL1, ultimately leading to changes in IMF content and fatty acid profile in Wandong cattle. While this study has elucidated the underlying reasons for the IMF differences in Wandong cattle, further validation is essential to delineate the specifics, thus establishing a novel theoretical framework for understanding the molecular regulation of lipid metabolism in ruminants.

## 4. Materials and Methods

### 4.1. Animals and Sample Collection

The experimental cattle in this study were sourced from smallholder farms in Fengyang County, Anhui Province, China, and consisted of purebred Wandong cattle. Thirteen animals were maintained in accordance with the Farmers’ Free Range Management program. Eight cattle with an average age of 3–4 years and a body weight of approximately 875.00 ± 40.09 kg were selected from the initial cohort. Based on IMF content determined by Soxhlet extraction, they were divided into a high-IMF group (HF, n = 4) and a low-IMF group (LF, n = 4). The cattle were fasted for 24 h before slaughter. The slaughtering process complied with the national standard operating procedures (GB/T 19477-2018, Cattle Slaughtering, China). During the slaughtering process, approximately 500 g of LD muscle samples were collected from the left side between the 12th and 13th ribs from each animal. Each sample was then divided into several portions for different analyses: a 20 g portion was sealed and stored at −80 °C for fatty acid determination; separate 10g portions were rapidly frozen in liquid nitrogen and stored at −80 °C for subsequent transcriptomic and metabolomic sequencing; the remaining samples were similarly pre-cooled in liquid nitrogen before transfer at −80 °C for preservation.

### 4.2. Lipid and Fatty Acid Contents in the LD Muscle

IMF content was quantified following GB 5009.6-2016 (Method I, National Food Safety Standard of China). Homogenized samples (1.00 ± 0.01 g) underwent Soxhlet extraction with anhydrous diethyl ether (85 ± 2 °C, 8 h). Fatty acid contents were analyzed by following GB 5009.168-2016 (Method III, National Food Safety Standard of China). Summarily, lipids were acid-hydrolyzed (8.3 mol/L HCl, N_2_, 70–80 °C, 40 min), ether-extracted, and converted to methyl esters via sequential saponification (0.2% NaOH-MeOH) and methylation (15% BF_3_-MeOH). Fatty acid methyl esters were separated on an HP-88 capillary column (100 m × 0.25 mm × 0.20 μm) with programmed heating (from 100 °C to 230 °C at 4° C/min), identified against certified standards, and quantified by peak-area normalization. Based on the IMF content, the samples were stratified into HF (17.42 ± 2.37) % content and LF (10.73 ± 2.37) % content groups for downstream analyses.

### 4.3. Transcriptome Sequencing and Analysis

Total RNA was extracted from LD using Trizol reagent (Invitrogen). To obtain a representative sample and minimize individual variation, LD muscles from four cattle in each group (HF and LF) were pooled and thoroughly homogenized to create one biological replicate. Total RNA was then extracted from this pooled homogenate, and three technical replicates (HF: H1, H2, H3; LF: L1, L2, L3) were generated in parallel for subsequent library construction and sequencing to ensure technical reliability. RNA integrity was verified spectrophotometrically (NanoDrop 2000 Thermo Fisher Scientific Co., Ltd., Madison, WI, USA). Sequencing libraries were prepared with mRNA enrichment, fragmentation, and cDNA synthesis, followed by adapter ligation and PCR amplification. Paired-end sequencing (150 bp) was performed on the Illumina NovaSeq X Plus platform (GeneDenovo Biotechnology Co., Ltd., Guangzhou, China). DEGs were determined using Omicsmart with thresholds of FC ≥ 1.2 and adjusted *p* < 0.05. Functional enrichment (GO, KEGG) and PCA were subsequently conducted.

### 4.4. Metabolomics Sequencing and Analysis

The samples (thawed at 4 °C) were extracted in a cold methanol/acetonitrile/water (2:2:1) solution, sonicated (30 min, 4 °C), incubated (−20 °C, 10 min), and then subjected to centrifugation (14,000× *g*, 20 min). Dried extracts were reconstituted in a mixture of acetonitrile and water (1:1) for HILIC-UHPLC analysis (HILIC column, 25 °C, 0.5 mL/min) using an acetonitrile/water gradient containing 25 mM ammonium acetate-ammonia. The initial composition of the gradient was 95% acetonitrile (0–0.5 min), decreasing to 65% at 7 min, then further decreasing to 40% at 9 min (maintained to 12 min) and returning to 95% at 12 min (maintained to 15 min). The high-resolution MS was conducted on an Orbitrap Exploris 48 instrument (GeneDenovo Biotechnology Co., Ltd., Guangzhou, China), operating at a resolution of 60,000 (m/z 70–1200) with ESI voltages set to +3500 V (positive) and –2800 V (negative). The XCMS-processed data were then subjected to K-nearest neighbor imputation, outlier removal, and total area normalization. The DEGs were determined by means of Omicsmart, with a defined VIP ≥ 0.8 and *p* < 0.05. The significance of KEGG pathway enrichment was determined by means of hypergeometric tests.

### 4.5. Joint Analysis of Metabolome and Transcriptome

Integrated metabolomic and transcriptomic analyses were performed using orthogonal partial least squares (O2PLS) and Pearson correlation analysis to characterize associations between the transcriptome and metabolome. All computations were conducted on the Omicsmart platform (https://www.omicsmart.com, accessed on 6 June 2025). Networks visualizing associations between DEGs and DAMs were also constructed using the same platform.

### 4.6. Statistical Analysis

Statistical analysis was performed using SPSS version 21.0, employing the independent two-sample *t*-test for IMF content and one-way analysis of variance (ANOVA) for fatty acid data. All data obtained from a sample size of n = 4 per group are presented as the mean and Standard Error of Mean (SEM). Significance was defined as a *p* < 0.05. For transcriptomic data, PCA was employed, while OPLS-DA was used for metabolomic data analysis. A model for visualizing the network relationships between DEGs and DAMs was constructed utilizing the Omicsmart platform.

## 5. Conclusions

This study revealed that variations in the IMF content and fatty acid profile in Wandong cattle, characterized by elevated levels of MUFAs such as C18:1n9c and C16:1, are closely linked to critical signaling pathways, including PPAR, AMPK, MAPK, and FoxO. The integrated metabolomic and transcriptomic analyses indicated that these pathways regulate essential lipid metabolic processes, including glycerophospholipid and fatty acid metabolism, by modulating the expression of key genes (SREBF1, ACSL1, SCD, FADS1), thereby collectively influencing IMF content. The findings of this study enhance our understanding of the molecular mechanisms underlying IMF formation in Wandong cattle. This knowledge provides a theoretical foundation and identifies promising candidate genes for molecular marker-assisted breeding.

## Figures and Tables

**Table 1:** Descriptive statistics of the IMF content and fatty acid profile of the Wandong cattle LD muscle.

Item	LF Group%	HF Group%	SEM	*p*-Value
IMF	10.73	17.42	2.37	0.031
C14:0	1.51	1.76	0.38	0.530
C16:0	20.70	24.00	2.62	0.255
C16:1	2.37	3.90	0.62	0.049
C17:0	0.72	1.09	0.30	0.267
C18:0	15.03	16.28	1.61	0.466
C18:1n9c	27.83	37.03	3.74	0.049
C18:2n6c	20.18	8.87	5.34	0.078
C18:3n3	1.27	0.53	0.22	0.015
C20:3n6	1.71	0.91	0.36	0.069
C20:4n6	8.23	4.81	1.90	0.122
C20:5n3	0.80	0.32	0.21	0.059
C18:3n6	0.39	0.20	0.07	0.041
SFAs	37.95	43.12	3.24	0.162
MUFAs	30.19	40.93	4.06	0.038
PUFAs	32.57	15.65	7.09	0.054

Note: IMF, intramuscular fat; SFAs, saturated fatty acids; MUFAs, monounsaturated fatty acids; PUFAs, polyunsaturated fatty acids. Fatty acids are noted as Cx:y (x, carbon number; y, double bond count). Data represent mean ± SEM (n = 4). Statistical analyses were performed with SPSS Statistics 21.0; differences were considered significant at *p* < 0.05 and highly significant at *p* < 0.01.

**Table 2:** Summary of transcriptome sequencing data of Wandong cattle.

Sample	Total Reads	Clean Reads	Mapped Reads	Q20%	Q30%	GC Content%
L1	45,722,634	46,077,682	43,858,794 (95.92%)	97.54	93.65	55.31
L2	46,260,976	46,727,756	44,499,981 (96.19%)	98.26	95.55	55.50
L3	48,466,588	48,851,988	46,552,884 (96.05%)	98.27	95.59	55.54
H1	42,850,892	43,032,774	40,962,113 (95.59%)	97.76	94.34	58.43
H2	57,277,954	57,556,550	54,780,512 (95.64%)	97.74	94.29	58.46
H3	53,428,120	53,737,138	50,993,802 (95.44%)	97.24	93.02	58.24

**Table 3:** KEGG pathways involved in the co-regulation of genes and metabolites.

Pathway	Metabolites	Genes
unsaturated fatty acid biosynthesis	oleic acid, arachidonic acid (peroxide free)	SCD, SCD5, ACOX1, ELOVL5, ELOVL6, and HSD17B12
fatty acid biosynthesis	C16:, C14:0, C18:1n9c	ACSL4, ACSL6, ACSBG1, ACSL1, OXSM, MCAT
fatty acid degradation	l-palmitoylcarnitine	ACSL3, ECHS1, HADHA, HADHB, ACADSB, ADH5, ACADM, ACSL6, GCDH, ALDH7A1, ADH6, ACAT1, CYP4A11, ACOX1, ACSL5, ACSBG1, EHHADH, ADH4, ACAA2, ACSL4, ACSL1, ACADL, CYP4A11
arachidonic acid metabolism	alongside prostaglandin a2, 5s-hydroxy-6e,8z,11z,14z-eicosatetraenoic acid, arachidonic acid (peroxide free)	HPGDS, PTGIS, PTGS2, CYP2E1, PTGDS, ALOX5, PTGES3, PTGES2, CYP2B11, CYP4F2, LTA4H, PLA2G2A, CYP2C21, Cyp2c23, GPX8, EPHX2, CYP4A11, PLA2G2A, CYP2J2, GPX7, PLA2G4A, PLA2G12A, CYP2U1, PLA2G2A, FAM213B, CYP4A1
glycerolipid metabolism	3-phospho-d-glycerate and glyceric acid	PL, AKR1B1, MOGAT1, GPAM, PPAP2C, MGLL, GK, AGPAT3, ALDH7A1, PNPLA2, LIPG, AGPAT2, AGK, LCLAT1, LPIN3, GPAT2, AGPAT5, LIPC, DGKB, DGKH, DGKE, MBOAT1, GLPK2, PPAP2A, PPAP2B, MBOAT2
glycerophospholipid metabolism	1-stearoyl-2-hydroxy-sn-glycero-3-phosphocholine, acetylcholine, choline, glycerophosphocholine, triethanolamine, and dl-serine	PCYT1A, PTDSS2, PLA2G15, GPAM, PPAP2C, CEPT1, GPD2, AGPAT3, PLA2G2A, PCYT2, LPCAT4, LCAT, CHPT1, AGPAT2, CRLS1, GPD1L, PLD1, LPCAT2, CDIPT, TAZ, ETNPPL, GPCPD1, LCLAT1, PLA2G2A, LPIN3, GPD1, PLA2G4A, GPAT2, PLA2G12A, AGPAT5, DGKB, DGKH, DGKE, GNPAT, LYPLA1, ACHE, ETNK1, LPGAT1, MBOAT1, CDS2, PLA2G2A, PPAP2A, PPAP2B, MBOAT2, PLD4

## Data Availability

The data presented in this study are available on request from the corresponding author.
